# Changes in GABA and glutamate concentrations during memory tasks in patients with Parkinson’s disease undergoing DBS surgery

**DOI:** 10.3389/fnhum.2014.00081

**Published:** 2014-03-07

**Authors:** Robert J. Buchanan, David P. Darrow, Kevin T. Meier, Jennifer Robinson, Dawn M. Schiehser, David C. Glahn, Zoltan Nadasdy

**Affiliations:** ^1^Division of Neurosurgery, Seton Brain and Spine InstituteAustin, TX, USA; ^2^Department of Psychology, University of Texas at AustinAustin, TX, USA; ^3^Department of Psychiatry, UT Southwestern Medical SchoolDallas, TX, USA; ^4^Department of Neurosurgery, University of Minnesota Medical SchoolMinneapolis, MN, USA; ^5^Department of Neurology, University of Utah School of MedicineSalt Lake City, UT, USA; ^6^Department of Psychology, Department of Electrical and Chemical Engineering, Department of Kinesiology, Auburn University MRI Research Center, Auburn UniversityAuburn, AL, USA; ^7^Department of Psychology, VA San Diego Healthcare System, Research ServiceSan Diego, CA, USA; ^8^Department of Psychiatry, Yale School of MedicineNew Haven, CT, USA; ^9^Department of Cognitive Psychology, Eötvös Loránd UniversityBudapest, Hungary; ^10^NeuroTexas Institute, St. David’s HealthCareAustin, TX, USA

**Keywords:** microdialysis, STN, Parkinson disease, implicit memory, DBS, GABA, glutamate, WPT

## Abstract

Until now direct neurochemical measurements during memory tasks have not been accomplished in the human basal ganglia. It has been proposed, based on both functional imaging studies and psychometric testing in normal subjects and in patients with Parkinson’s disease (PD), that the basal ganglia is responsible for the performance of feedback-contingent implicit memory tasks. To measure neurotransmitters, we used *in vivo* microdialysis during deep brain stimulation (DBS) surgery. We show in the right subthalamic nucleus (STN) of patients with PD a task-dependent change in the concentrations of glutamate and GABA during an implicit memory task relative to baseline, while no difference was found between declarative memory tasks. The five patients studied had a significant decrease in the percent concentration of GABA and glutamate during the performance of the weather prediction task (WPT). We hypothesize, based on current models of basal ganglia function, that this decrease in the concentration is consistent with expected dysfunction in basal ganglia networks in patients with PD.

## Introduction

Parkinson’s disease (PD) has been the most widely studied movement disorder. Various modalities have been used to quantify the dysfunction of both the nigrostriatal and the mesocortical systems in PD. Although a variety of currently available “non-invasive” methods such as fMRI, Positron Emission Tomography, Single-photon Emission Computed Tomography (SPECT) and most recently DaTscan imaging allow for measuring changes in brain metabolism in relationship to PD (Niethammer et al., 2012), these changes are linked indirectly to transmitter release. Moreover, these neuroimaging methods have the limitation of being more “qualitative” than “quantitative”. In contrast, invasive techniques such as microdialysis provide direct and quantitative measurements of the chemical constituents of fluid in the extracellular space of the brain. Neurosurgery provides an opportunity to safely insert a microdialysis catheter into a region of interest enabling researchers to study task-contingent modulation of neurotransmitters in the human brain in patients undergoing neurosurgical intervention (Fried et al., 1999). We implemented microdialysis in deep brain stimulation (DBS) surgeries, an increasingly common treatment option for patients with PD. The measurement of both glutamate and GABA in this study provides a neurochemical atlas of subthalamic nucleus (STN) function in patients with PD. Our results can be directly correlated with future functional neuroimaging studies of STN metabolism during implicit memory tasks in both normal subjects and PD patients.

**Figure 1:**
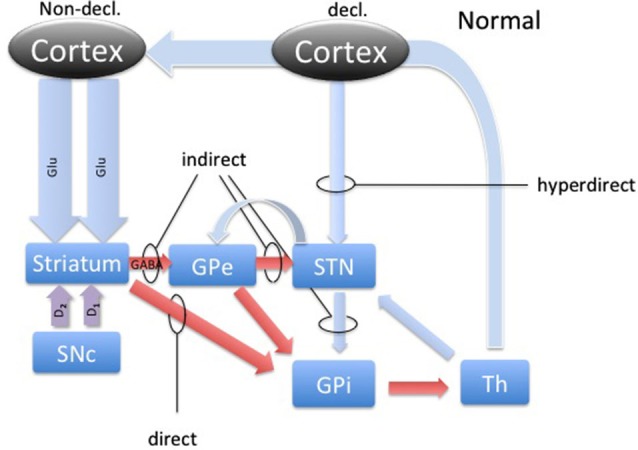
**The basal ganglia circuitry.** The scheme represents the main pathways of the BG in the healthy brain with respect to declarative and implicit memory functions. Blue arrows represent glutamatergic, red arrows GABAergic and magenta arrows represent dopaminergic connections. Direct, indirect and hyperdirect pathways are indicated.

DBS involves placement of electrodes into specific regions of the brain, allowing the delivery of electrical impulses, and recently was shown to be one of the best treatment options available for individuals with advanced PD (Weaver et al., 2009). A microdialysis catheter can be placed in this same region as a DBS electrode, providing an opportunity for researchers to examine the neurotransmitter correlates of memory function in PD, without any additional damage to brain tissue. This study using intraoperative-microdialysis allowed for the first time in the human brain (i) to assess the modulation of transmitter release during declarative and implicit memory tasks; and (ii) study neuro-pharmacological effects of PD on memory functions. Nevertheless, the interpretation of such data requires a good understanding of the neural circuitry and, based on the well-established scheme of direct, indirect and hyper-direct pathways of the basal ganglia (BG) system (Figure 1) we were able to make assumptions about the neurotransmitter levels during an implicit memory task performed by normal subjects (Figure 3). This assumption was used to predict changes in neurotransmitter levels in the STN under PD assuming correlation with its known effect on implicit memory performance. Nevertheless, because molecular samples cannot be ethically collected from a normal population, the net contribution of the pathology to the neurochemistry of BG could not be completely assessed. Therefore, predictions concerning the neurotransmitter release in the healthy BG during implicit memory tests could not be tested.

**Figure 2:**
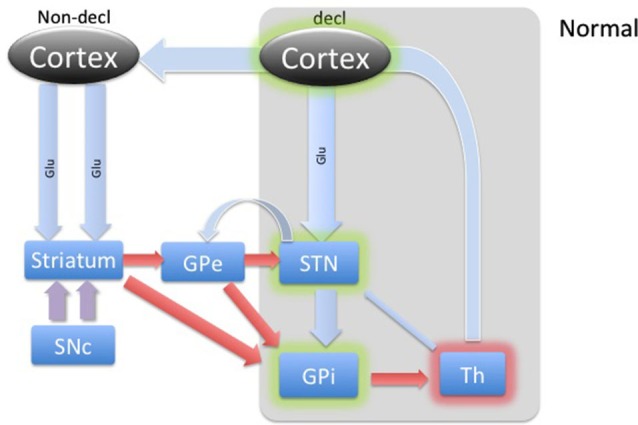
**Diagram of cortical and BG activity during a declarative memory task in a healthy control.** The thickness of arrows represents the theoretical expected concentration change relative to the baseline depicted in Figure 1. The gray-shaded area represents the part of the BG circuitry involved in declarative memory task. Green shadows represent activation and the red shadow represents suppression of the structures.

**Figure 3:**
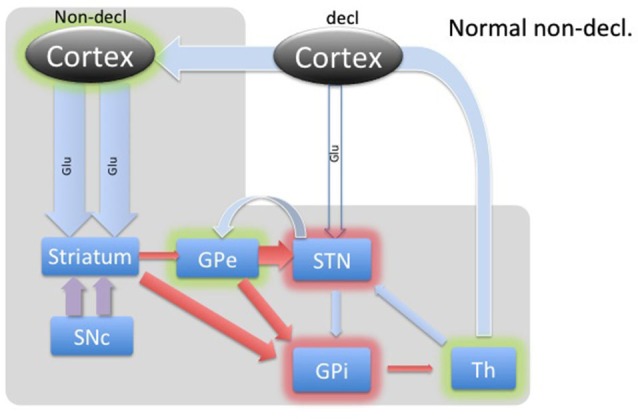
** Diagram of cortical and BG activity during an implicit memory task in a healthy control.** The expected changes in STN are the increased concentration of GABA and the decreased concentration of glutamate. The thickness of arrows represents the theoretical expected concentration change relative to the baseline depicted in Figure 1. The gray-shaded area represents the part of the BG circuitry involved in implicit memory task. Green shadows represent activation and red shadows represent suppression of activity in the structure.

The activity dynamics in the BG is best understood in terms of excitation, inhibition and disinhibition (Figure 1). The most prevalent excitatory and inhibitory neurotransmitters in the BG are glutamate (GLU) and gamma-amino butyric acid (GABA; Sian et al., 1999). In this study, we took microdialysate from five right handed patients with PD during DBS placement in the non-dominant (right) STN and later analyzed it for the concentrations of glutamate and GABA. Intraoperatively, patients participated in cognitive testing during the collection of the microdialysis samples. Patients performed the weather prediction task (WPT) with immediate feedback, known to engage implicit memory, and word recollection tasks to engage declarative memory (Knowlton et al., 1994). We hypothesized that there would be a change in the relative concentrations of these neurotransmitters in the STN during the WPT relative to declarative memory task, and this change is opposite of what is expected to be in healthy subjects, representing involvement of the STN in this form of implicit memory processing and its pathology.

## Parkinson’s disease (PD) and learning/memory

PD patients have significant deficits on tests of learning and memory. These deficits are most severe in implicit memory tasks (Packard and McGaugh, 1992; Knowlton et al., 1996). The anatomical substrates of declarative and implicit memory have been and continue to be studied. The central role of the basal ganglia in implicit memory has been further supported with the WPT when the performance of amnesic patients and Parkinson’s patients were compared as they performed memory tasks (Gluck and Bower, 1988; Poldrack et al., 2001). Parkinson’s patients had significant difficulties with the WPT while amnesic patients displayed difficulties recalling “facts” about the same task. As the pathology in PD lies within the basal ganglia (dopamine producing cells of substantia nigra), it was concluded that the basal ganglia is involved in implicit memory (Knowlton et al., 1996). This hypothesis has been supported using fMRI to show increased activity in the basal ganglia during motor memory or habit learning (Rauch et al., 1997; Poldrack et al., 1999). The WPT, originally thought to engage only implicit memory, has been used in many studies and explored to further understand its cognitive engagement. The performance of PD patients on the WPT was refined further through experiments suggesting that performance is impaired only when feedback from responses are provided and if such feedback is provided immediately (<6 s) (Shohamy et al., 2004a; Foerde and Shohamy, 2011). For example, if the WPT is modified by removing trial-by-trial feedback or if the feedback is delayed, PD patients perform near controls. Additionally, evidence suggests that the medial temporal lobe (MTL) is engaged early during WPT in healthy controls and PD patients, diminishing rapidly with continued training (Poldrack et al., 2001; Moody et al., 2004).

In retrospect, it is not surprising that the WPT requires declarative memory to some extent, especially at the start of a new task. The failure of most PD patients to transition from a MTL mode of memory to a BG mode still leaves many questions unanswered concerning the interaction between these two modes of memory and their control (Foerde and Shohamy, 2011). While current research has not clarified the pattern of interaction between BG and MTL modes of memory, evidence exists to support model of competition and cooperation depending on the specific task (Poldrack et al., 2001; Dickerson et al., 2011).

**Figure 4:**
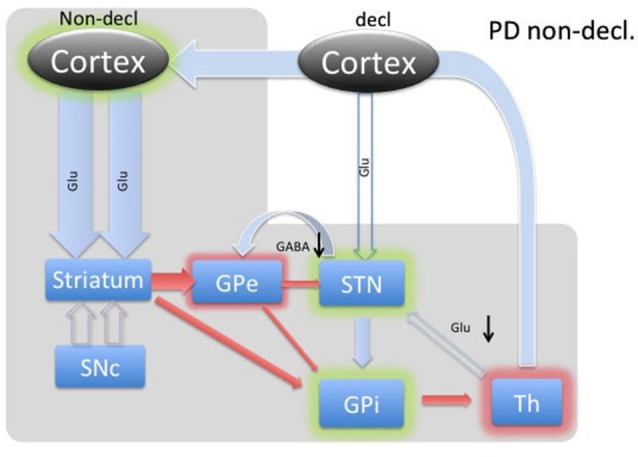
**Diagram of cortical and BG activity during an implicit memory task in a Parkinson’s patient.** The expected changes in STN are the decreased concentration of GABA and the increased concentration of glutamate. The meanings of arrows and shadows are the same as in Figure 3.

The BG’s involvement in implicit memory is further corroborated by the fact that DBS stimulation in STN differentially affects declarative and implicit memory performance by impairing declarative memory, whereas improving implicit memory (Hälbig et al., 2004). Hence, the WPT task should elicit a strong response in the healthy circuitry of STN (Figure 3) and is expected to be reversed in PD (Figure 4), explaining the implicit memory deficit. With the lack of direct neurochemical evidence in support of the above hypothesis we performed microdialysis in the STN on PD patients during WPT.

Most studies of implicit memory have emphasized the role of the caudate, globus pallidus, and putamen, but the STN has not been extensively studied, despite it being recognized as a critical structure within the basal ganglia (Redgrave et al., 2010). The STN is primarily composed of neurons containing glutamate. It contains sub-regions within, organized regionally, that mediate limbic, associative, and sensory-motor functions. It is the ventro-medial regions that comprise the limbic and associative territories (Parent and Hazrati, 1995; Shink et al., 1996; Joel and Weiner, 1997). The dorsal-lateral aspect of the STN is targeted with DBS to treat the motor symptoms (tremor, rigidity and bradykniesia) of PD. In newer models of BG connectivity the STN boasts efferent connections to at least the Internal Globus Pallidus (GPi) and Substantia Nigra pars reticulata (SNr), Substantia Nigra pars compacta (SNc) and Ventral Tegmental Area (VTA), and the Globus Pallidus Externa (GPe; Redgrave et al., 2010). Additionally, it receives projections from the GPe, SNc and VTA, thalamus, and the cortex (Redgrave et al., 2010).

Microelectrode recordings from patients with PD have revealed that the STN is hyperactive, with baseline firing rates of 35–50 Hz, and exhibits abnormal synchronized oscillations (Hutchison et al., 1998; Levy et al., 2002; Sterio et al., 2002). Hyperactivity of the STN in PD has become a hallmark of present models of basal ganglia dysfunction in PD, where the lack of dopamine causes the STN to fire without a functional “brake” (GABA) from the GPe through the indirect pathway (Figure 4; Smith et al., 1990; Bevan et al., 1997, 2007). These effects are theorized to produce the commonly seen signs and symptoms of PD, such as bradykinesia and akinesia. Additionally, the STN has a unique position in BG function by receiving direct cortical (Glu) input via the “hyperdirect” pathway (Figure 1). This allows for a rapid response of the STN to excite the pallidum, which would further inhibit the thalamus (Nambu et al., 2002).

It is now well established that dopamine plays a prominent role in learning by modulating excitatory and inhibitory cortico-striatal projections. Facilitating this dynamic are the individual dopamine receptors, D1 and D2. D1 is known to be expressed predominantly on the medium spiny neurons (MSN) in the striatum that project to the GPi, comprising the direct pathway (Figure 1). D2 expression is relegated to the MSNs that project to the GPe, which is recognized as the indirect pathway (Figure 1). Additionally, these cells are also known as Go and No-Go cells, respectively, due to the function of the direct pathway to disinhibit and the indirect pathway to further inhibit the GPi from the striatum. Through its effects on Go and No-Go cells in the striatum, evidence is mounting that DA increases the signal-to-noise ratio of striatal cells excited by cortico-striatal projections, essentially highlighting rewarding associations (Nicola et al., 2000). Additionally, dips in tonic levels of DA provide a mechanism of weakening such relationships, dissociating unrewarding associations (Clark et al., 1987).

Computational research is beginning to tackle the precise role of this circuitry in implicit memory formation by explaining how pathological loss of dopaminergic neurons leads to implicit memory dysfunction in PD patients (Frank, 2005). Previous studies have demonstrated an unusual effect of DA-replacement therapy in PD patients (Cools et al., 2001). While “unmedicated” patients were found to perform poorly on implicit memory tasks and nearly normally on explicit memory tasks, medicated patients were found to perform better on implicit memory tasks but worse on explicit tasks. As suggested by computational modeling, the reduced dynamic range of DA is at least somewhat responsible for the underlying pathology (Frank, 2005).

Accordingly, through an updated model of BG circuitry shown in Figure 1, we attempted to predict the neurochemical status of the STN during explicit and implicit memory formation in PD patients. Through the hyperdirect pathway, the STN receives glutamatergic input from temporal/prefrontal cortical areas (Figures 1 and 2) associated with the declarative memory. This excitatory input may function to keep the glutamate concentration in the STN higher during declarative memory tasks than it is during implicit tasks. Proposed characterization of the hyperdirect pathway provides at least two possible effects on relative levels of Glu during the WPT and declarative tasks. The short temporal nature might prove difficult to detect relative changes in concentration during microdialysis (Nambu et al., 2002). Another possibility is that because PD patients are known to utilize declarative memory even when presented with tasks that are more optimally completed with implicit memory, either due to inability or compensation, it is possible that the hyperdirect pathway frequently engages the STN, which would persistently reinforce declarative memory (Shohamy et al., 2004b). Chronic engagement of the MTL, and corresponding inhibition of STN, might provide a milieu of relatively excessive Glu. Enforcing an implicit memory task could provide a short respite to glutamatergic excitation. Additionally, the level of GABA is expected to be slightly higher or the same during declarative memory, which is mostly driven by the recently discovered feedback control from the GPe (Nambu et al., 2000).

During implicit memory tasks, the BG is driven by an input from a corticofugal system that is anatomically and functionally distinct from the declarative memory system (Figure 3). The corticofugal input derives from the posterior orbitofrontal, anterior insular cortex as well as the posterior medial prefrontal anterior cingulate cortex that project exclusively to striosomes in medial and ventral caudate nucleus (Eblen and Graybiel, 1995). Normal engagement of a task requiring implicit memory should disinhibit thalamic activity through the direct pathway and release activation of the GPi through the indirect pathway. In this way, the expectation is that the STN will experience increased GABAergic stimulation by the GPe and increased Glu via proportional feedback from thalamic projections in healthy patients. In PD patients, we predict that the lack of potentiation by the SNc results in abnormal hyperactivity of the No-Go MSNs from the striatum to the GPe, resulting in a hyperactive STN and reduced glutamatergic feedback from the thalamic projections. Thus, we expected to find reduced GABA and Glu in the STN during the WPT in PD patients.

## Materials and methods

### Patient selection

Five patients (*n* = 5) with medically refractory PD or those who experienced intolerable side effects from medications are appropriate candidates for DBS. Patients underwent a comprehensive neuropsychological evaluation to rule out dementia and psychiatric disease. Those without contraindications for DBS surgery were told of the risks and benefits of the DBS operation. Patients who decided to proceed with the operation were told of the Institutional Review Board (IRB) approved intra-operative study. Informed consent was obtained. Prior to surgical intervention, patients were briefed about the study and told that they could withdraw from the research at any time without any penalty or consequence. Approval was obtained from the local Institutional Review Board.

### Deep brain stimulation procedure

Twenty-four hours prior to DBS surgery, patients discontinued all medications used to treat their PD. Deep brain stimulation procedures were performed stereotactically (Medtronic-NeXframe) by the same neurosurgeon, author Robert J. Buchanan (RJB). Patients received a high-resolution 3T MRI scan with a stereotactic bone/skull fiducial system. The coordinates of the human STN were established in three-dimensional Cartesian coordinate system, x, y and z. Stereotactic targeting of the right STN was accomplished with a combination of atlas coordinates (“indirect method”) and the patient’s MRI images (“direct method”), in addition to real-time single cell recordings intra-operatively. In the operating room, the patient was placed in a comfortable sitting/lounge chair position on the operating table. A short-acting sedative (propofol) was administered. The patient was then covered with blankets to maintain ambient temperature (98.6°C). The entry points of the skull were determined with the use of the NeXframe System. The areas were infiltrated with 1% lidocaine and an incision was made in a curvilinear fashion. A right-sided burr hole was then made in the skull with the use of a high-speed pneumatic drill. The NeXframe (Medtronic) was attached to the patient’s skull, and then registered to the frameless image guided stereotactic system (BrainLab). The cannula and microelectrode were placed within the “Microelectrode drive”, which lowers the electrode by millimeter fractions into the brain towards the STN. The microelectrode provided the team with single cell recordings from brain regions during the descent to the target (STN). An array of three microelectrodes was used to somatotopically map the STN. During the microelectrode recordings, propofol was discontinued. The dorsolateral part of the nucleus represents the motor territory and is the surgical DBS target. The microelectrode track that revealed the longest (at least 5 mm of STN cell activity) was chosen for placement of the DBS electrode. The microelectrode track that was most medial and ventral to that track, which also clearly had STN cell activity, was chosen for the microdialysis experiment. The ventral and medial regions of the STN are likely to be association and limbic areas, respectively, rather than motor (Parent and Hazrati, 1995; Middleton and Strick, 2000). The microdialysis catheter (CMA, Boston) was placed into the STN based on techniques and coordinates described previously.

After the STN target was located and catheter placement accomplished, there was a 45 min period of microdialysis “wash-out” to achieve steady state baseline measurements of GABA and GLU. Next, neurotransmitter samples were obtained every 5 min while patients were administered cognitive tasks. Tasks were separated by one sample collection each. Following the completion of the research protocol, the microdialysis catheter was removed and the DBS procedure was completed as per usual.

### Microdialysis

The microdialysis equipment was made by CMA Microdialysis Corporation, specially designed for use in human brain tissue in clinical settings. The outer diameter of the shaft of the probe is 0.67 mm. The diameter of the microdialysis membrane is 0.5 mm. Both of these diameters are smaller than the diameter of the standard surgical cannula (1.3 mm) that is used to carry the microeletrode down to the target. The cannula has been custom designed to accommodate the outer diameter of the deep brain-stimulating electrode, which is 1.27 mm. The length of the catheter is 200 mm and the length of the dialysis membrane is 5 mm. The microdialysis catheter was placed into the STN of the non-dominant hemisphere (right side in all cases) following a previously made track.

The microdialysis pump device (CMA 107) was set at a flow rate of 5 ul/min for this experiment. The CMA 142 “Microfraction Collector” was used to collect the dialysate. All equipment was supplied by the manufacturer in a sterile fashion. The sterile pyrogen free PBS solution was used as the perfusate contained in grams/liter, NaCL 8, KCL 0.2, CaCL2 × 2 20 0.132, MgCl2 × 6 H20 0.1, Na2HPO4 1.15, KH2PO4 0.2 at a pH of 7.4. The levels of the desired neurotransmitters in the dialysate were analyzed by high performance chromatography (HPLC). Our technology enabled measurements of the neurotransmitters to be made in the nmol/L to micromole/L range.

### Quantification of GABA and glutamate

GABA and Glutamate were quantified using isocratic HPLC with electrochemical detection. Briefly, microdialysis samples collected as described above were mixed with a derivatizing reagent to prepare o-phthaldehyde (OPA) derivatives of GABA, glutamate, and homoserine (internal standard). The mixture was prepared using an ESA 542 autosampler exactly 1 min before injection into the HPLC system (20 μl of dialysate and 15 μl of working derivatizing reagent). The derivatizing reagent contained 27 mg of OPA dissolved in 1 ml methanol, 9 ml of 100 mM sodium tetraborate and 5 μl of β-mercaptoethanol. Twenty-seven μl of final sample were injected into the HPLC. Glutamate, homoserine, and GABA eluted at 2.0, 5.1, and 14.6 min, respectively. GABA and glutamate were quantified by comparing peak area ratios of these compounds to homoserine against the linear regression of ratios of a set of calibrators from 0.1 to 500 ng/ml. The concentration of GABA and glutamate was expressed in ng/ml.

The mobile phase consisted of 27%(v/v) methanol, 50 μM ethylenediamine tetra-acetic acid (EDTA), 75 mM phosphoric acid (pH 6.75 with NaOH), and Milli-Q water. The flow rate of the mobile phase was 1 ml/min and the column temperature was 30°. The HPLC system consisted of an ESA 584 pump, ESA 542 autosampler, Spherisorb C18 column (3 μ, 4.6 × 100 mm), and a Coulochem III detector equipped with a 5014B detector cell. The detector was set at 150 mv on E1, 550 mv on E2, and included a guard cell set at 650 mv.

### Declarative memory task

Patients performed a verbal learning task in which they were read a list of words, and asked to repeat them back. The list was repeated several times. Following the implicit memory task (approximately 20 min later), the patients were asked to recall the list of words read to them prior to the implicit memory task (delayed recall).

### Implicit memory task

Patients performed an implicit memory task previously described in the literature (Knowlton et al., 1996; Moody et al., 2004). Patients were oriented to the task prior to administration. The task is frequently referred to as the WPT/Game (Gluck and Bower, 1988). There are four cues in the task, which are represented by cards with geometric shapes, and these cues predict one of two outcomes approximately 60–85% of the time. Subjects see a set of cues on a computer screen and their task is to “guess” whether the cues predict “sun” or “rain”. Each of these cues is independently and probabilistically related to the outcome, and the two outcomes occur equally often. If they make a correct response they hear a “high” tone and see a smiling face, and if they predict incorrectly they hear a “low” tone and see a frowning face. The probabilistic nature of this task appears to encourage optimal play through an implicit pathway and likely parallels the gradually acquired habit learning tasks that are performed by experimental animals. The total task time was approximately 10 min. We calculated the total scores from the first to last trial (maximum 100 trials) in the subject’s average score.

### Statistical analysis

Paired *t*-tests comparing the percent change of GLU and GABA levels from a baseline during the implicit and declarative memory task were performed, where baseline was computed as the average of the previous and subsequent measurements taken at rest. SAS version 9.2 was used for all calculations.

## Results

It has been demonstrated in the literature that patients with PD have difficulty learning the WP task (Knowlton et al., 1996). Our patients followed the same trend (Table 1). As a whole, our patients scored 52.8%, no better than chance and significantly worse than the healthy average (68.2%, Knowlton et al., 1994 and 62.8%, Gluck et al., 2002). The difference relative to age matched healthy control was significant no matter which study we are comparing it to (mean = 68.2%; SD = 7.36; Student *t*_(39,5)_ = −4.503; *p* < 0.001 (Knowlton et al., 1994); mean = 62.8%; SD = 7.2, difference: Student *t*-test *t*_(32,5)_ = −2.875; *p* = 0.007) (Gluck et al., 2002).

**Table 1:** **Nondeclarative task (ND) and declarative memory recall test (D1, D2) performance by patient in percentage correct**.

Patient	ND	D1: Trial 1	D1: Trial 2	D1: Trial 3	D2 (20 min)
**1**	61.9%	42%	58%	42%	25%
**2**	62.0%	25%	58%	83%	67%
**3**	54.0%	58%	92%	75%	75%
**4**	43.0%	33%	75%	58%	58%
**5**	43.2%	67%	67%	58%	75%
**Average**	52.8%	45%	70%	63%	60%

The results of the subjects during the declarative memory task are shown in Table 1. Patients averaged 59% recall overall during the first three trials (D1) and 60% at 20 min (D2). There were no statistically significant differences.

**Figure 5:**
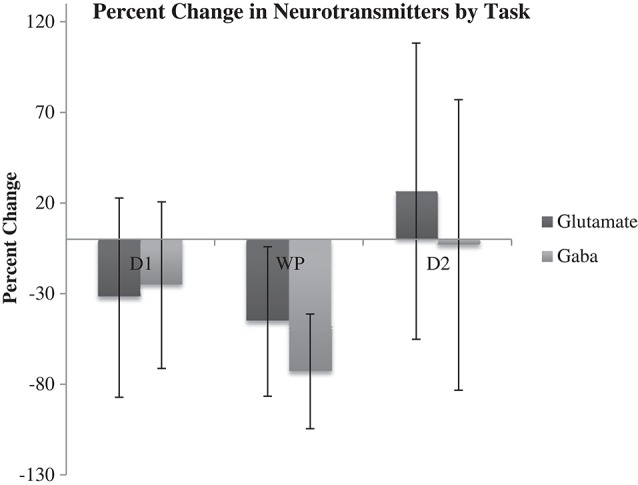
**Average percent change of Glu and GABA from baseline during declarative tasks (D1, D2) and WTP with 95% confidence intervals**.

Results of microdialysis during each of the tasks in their order of sequence are depicted in Figure 5 with 95% confidence intervals shown in Table 2. During the first and second declarative tasks, the percent change of glutamate and GABA concentrations from baseline (computed as the average of prior and subsequent concentrations) demonstrated no statistically significant difference, separately or when combined. In contrast, the percent change of concentrations of glutamate and GABA were found to be lower during the WPT (*P* < 0.038 and *P* < 0.0031). The change during the WPT was 30 and 55% of baseline for glutamate and GABA, respectively.

**Table 2:** **95% confidence intervals of percent change of neurotransmitter from baseline for each memory test**.

Test	Glutamate	GABA
**D1**	(−87.12, 22.79)	(−71.25, 20.68)
**WP**	(−86.60, −4.07)	(−104.50, −41.23)
**D2**	(−55.11, 108.30)	(−83.29, 77.05)

## Discussion

### Declarative task

During the declarative memory task, we hypothesized that glutamate released from the cortical regions (via hyperdirect pathway) would be expected to increase from baseline levels to stimulate the STN in healthy subjects, as illustrated in Figure 2. The input from cortical levels would potentiate the indirect pathway, dampening the basal ganglia (Aron and Poldrack, 2006). This is a cognitive version of “blocking” an initiation of the “Go” response. STN excitation would lead to a stimulation of the GPi. The GPi, in turn, would cause further inhibition of those regions of the thalamus anatomically connected to BG memory systems. Additionally, while overall activity in the STN is predicted to be relatively increased, the exact contribution from inhibitory and excitatory input is less clear. With newer models of BG circuitry highlighting feedback from the STN to the GPe, we predicted mild dampening of the STN by the GPe (Redgrave et al., 2010). Acknowledging tremendous uncertainty about the overall dynamics in the rest of the BG during declarative memory formation in healthy adults, we cautiously predicted relatively increased or unchanged GABA within the STN during a declarative memory task in a healthy control.

In PD patients, reduced dopaminergic input to the striatum provides baseline hyperactivity of the STN. During a declarative memory task, the hyperdirect pathway would function as in healthy patients by providing increased glutamatergic input, but compensatory engagement of the declarative memory system might wash-out any marginal increase. The effect of maintaining or engaging declarative memory on the GPe, and thus the STN, is unclear. In our patients, we found no noticeable average percent change in Glu or GABA from baseline during either declarative memory task.

Washout from compensatory engagement of the MTL is one possible explanation for our findings. This could be visualized as asking a patient already engaging the MTL to continue to engage the MTL. We observed large variation in Glu, which could be due to a variety of forces. Our analysis of newer models of BG circuitry does not highlight the importance of GABA activity in the STN during declarative memory. This may explain our finding of no significant change in GABA, where it may take on a wide range of values without direct effect.

### Implicit task

In contrast, the WPT activates the cortico-striatal pathway, which converges with the nigro-striatal pathway from SNc. SNc input enables feedback-contingent learning by immediate D2 release to reinforce habit-memory (Schultz, 2013). The two inputs cause a balanced moderate inhibition of the GPe, which in turn inhibits STN. The STN reduces excitation of the GPi, which disinhibits the thalamus. Additionally, these combined inputs would facilitate a release of the thalamus through the direct pathway, illustrated in Figure 3. Based on this model, we would expect dampening of the STN during implicit memory tasks in healthy patients. Specifically, we theorize that the GABA concentration within the STN would increase as a result of cortical stimulation of the striatum and subsequent inhibition of the GPe. This would remove the STN’s stimulatory effect on the GPi to further inhibit thalamo-cortical circuits. The net effect would be activation or disinhibition of thalamocortical circuits.

In PD patients during an implicit memory task (Figure 4), the lack of dopaminergic input does not provide the previously described selective amplifying effect to the excitatory thalamo-cortical projections. Thus, cortico-striatal excitation provides nonselective stimulation, resulting in further inhibition of the GPe through the indirect path and, most likely, a much smaller amount of inhibition of the GPi through the direct pathway. In turn, GABA was predicted to be decreased in the STN. The GPi would experience the summative effect of inhibition from the striatum and GPi as well as diffuse stimulation from the STN. Since the STN is already hyperactive, we predicted any marginal increase in activity would dominate the inputs of the GPi, resulting in inhibition of the thalamus.

Thalamic inhibition by the GPi would release excitatory efferent projections to the STN, reducing glutamate. Simultaneously, compensatory hyperdirect stimulation of the STN would cease, possibly also reducing the relative glutamate concentration. Our data demonstrates reduced levels of GABA and glutamate during the WPT, which is in agreement with our predictions.

### Limitations

As our understanding of BG circuitry and dynamics continues to evolve, the interdependence of sub-cortical and cortical connectivity and interaction in the context of learning and memory makes experimental predictions difficult and uncertain. In light of this dynamic field, our predictions for the relative concentrations of GABA and glutamate within the STN are only hypotheses. While our experimental setup with the WPT did produce statistically significant changes, the temporal limitations of microdialysis and a more nuanced relationship between implicit memory and the WPT only allow the suggestion of a causal relationship and its underlying dynamic.

### Future directions

In light of these findings, we plan to update our methodology to examine more specific facets of the neurochemical basis of habit learning in PD patients. For instance, whether or not immediate-feedback contingent neurochemical changes during implicit memory tasks corroborate the behavioral deficit that is observed when PD patients are performing WP task needs to be further tested. We hope to be able to provide clarity between the pathological basis of PD and BG dynamics. Newer technological methods may also improve some of the limitations of microdialysis alone and provide mechanisms for rigorous consistency between overlapping fields, such as memory and learning.

### Summary

In summary, through the use of microdialysis, we examined the relative concentrations of GABA and glutamate within the STN during declarative memory and feedback-contingent implicit memory tasks in PD patients. We predicted changes in neurotransmitter levels within the STN based on newer models of BG circuitry and a current understanding of Parkinson’s pathology. We found no statistically significant changes in GABA or glutamate during declarative memory tasks, but we did observe statistically significant reductions in GABA and glutamate during completion of the WPT. We provide these results as the first attempt to bring neurochemical measurements of the basal ganglia to compare and update current computational and circuit models of functional activity in Parkinson’s patients.

## Conflict of interest statement

The authors declare that the research was conducted in the absence of any commercial or financial relationships that could be construed as a potential conflict of interest.
